# The function of the gut microbiota–bile acid–TGR5 axis in diarrhea-predominant irritable bowel syndrome

**DOI:** 10.1128/msystems.01299-23

**Published:** 2024-02-08

**Authors:** Kai Zhan, Haomeng Wu, Yongyin Xu, Kehan Rao, Huan Zheng, Shumin Qin, Yuanming Yang, Rui Jia, Weihuan Chen, Shaogang Huang

**Affiliations:** 1 Dongguan Hospital of Guangzhou University of Chinese Medicine, Dongguan, China; 2 The Second Clinical College of Guangzhou University of Chinese Medicine, Guangzhou, China; 3 State Key Laboratory of Dampness Syndrome of Chinese Medicine, The Second Affiliated Hospital of Guangzhou University of Chinese Medicine, Guangzhou, China; 4 Collaborative Innovation Team of Traditional Chinese Medicine in Prevention and Treatment of Functional Gastrointestinal Diseases, Guangzhou University of Chinese Medicine, Guangzhou, China; 5 Science and Technology Innovation Center, Guangzhou University of Chinese Medicine, Guangzhou, China; 6 The First School of Clinical Medicine, Guangzhou University of Chinese Medicine, Guangzhou, China; Vanderbilt University Medical Center, Nashville, Tennessee, USA

**Keywords:** diarrhea-predominant irritable bowel syndrome, gut microbiota, bile acid, TGR5

## Abstract

Imbalanced gut microbiota (GM) and abnormal fecal bile acid (BA) are thought to be the key factors for diarrhea-predominant irritable bowel syndrome (IBS-D), but the underlying mechanism remains unclear. Herein, we explore the influence of the GM–BA–Takeda G-protein-coupled receptor 5 (TGR5) axis on IBS-D. Twenty-five IBS-D patients and fifteen healthy controls were recruited to perform BA-related metabolic and metagenomic analyses. Further, the microbiota-humanized IBS-D rat model was established by fecal microbial transplantation (FMT) to investigate the GM–BA–TGR5 axis effects on the colonic barrier and visceral hypersensitivity (VH) in IBS-D. Finally, we used chenodeoxycholic acid (CDCA), an important BA screened out by metabolome, to evaluate whether it affected diarrhea and VH via the TGR5 pathway. Clinical research showed that GM associated with bile salt hydrolase (BSH) activity such as *Bacteroides ovatus* was markedly reduced in the GM of IBS-D, accompanied by elevated total and primary BA levels. Moreover, we found that CDCA not only was increased as the most important primary BA in IBS-D patients but also could induce VH through upregulating TGR5 in the colon and ileum of normal rats. TGR5 inhibitor could reverse the phenotype, depression-like behaviors, pathological change, and level of fecal BSH in a microbiota-humanized IBS-D rat model. Our findings proved that human-associated FMT could successfully induce the IBS-D rat model, and the imbalanced GM–BA–TGR5 axis may promote colonic mucosal barrier dysfunction and enhance VH in IBS-D.

**IMPORTANCE:**

Visceral hypersensitivity and intestinal mucosal barrier damage are important factors that cause abnormal brain–gut interaction in diarrhea-predominant irritable bowel syndrome (IBS-D). Recently, it was found that the imbalance of the gut microbiota–bile acid axis is closely related to them. Therefore, understanding the structure and function of the gut microbiota and bile acids and the underlying mechanisms by which they shape visceral hypersensitivity and mucosal barrier damage in IBS-D is critical. An examination of intestinal feces from IBS-D patients revealed that alterations in gut microbiota and bile acid metabolism underlie IBS-D and symptom onset. We also expanded beyond existing knowledge of well-studied gut microbiota and bile acid and found that *Bacteroides ovatus* and chenodeoxycholic acid may be potential bacteria and bile acid involved in the pathogenesis of IBS-D. Moreover, our data integration reveals the influence of the microbiota–bile acid–TGR5 axis on barrier function and visceral hypersensitivity.

## INTRODUCTION

Irritable bowel syndrome (IBS) is a common functional gastrointestinal disease; diarrhea-predominant irritable bowel syndrome (IBS-D) is the main subtype of IBS, accounting for 46%–62% of the total number of IBS (1). The main symptom of IBS-D is diarrhea, accompanied by chronic recurrent abdominal pain and changes in bowel habits (2). IBS is known as one of the most expensive gastrointestinal diseases due to the characteristics of no specific drugs and easy recurrence of symptoms (3). Therefore, the development of safe and effective drugs for treating IBS-D has vital social and economic significance.

The exact pathogenesis of IBS-D is incompletely understood. Recently, an increasing number of studies have demonstrated that the gut microbiota (GM)–bile acid (BA) axis plays an important role in the pathological mechanism of IBS-D (4, 5). At the phylum level, the proportion of *Firmicutes* is significantly reduced, and that of *Bacteroides* is increased in IBS-D patients (6). At the genus level, the abundance of *Lactobacillus*, *Weissella*, and *Parasutterella* was significantly decreased, with a corresponding increase in *Faecalitalea, Escherichia/Shigella,* and *Fusobacterium*, which is a recurring finding, especially in IBS-D patients (7, 8). Meanwhile, after the treatment of IBS-D patients with microecological agents, the imbalanced GM was adjusted, abdominal pain and diarrhea were significantly reduced, and even depression or anxiety was relieved (9, 10).

GM imbalance will inevitably lead to changes in its metabolism. BAs were one of the important substances in GM metabolism. Abnormal BA metabolism is one of the common phenomena in IBS-D patients (11), which is mainly characterized by the increase of fecal primary BA (12). Chenodeoxycholic acid (CDCA) is one of the vital primary BA, which has been confirmed to be increased in IBS-D (13). Moreover, the administration of the oral physiological dose of CDCA to healthy volunteers can accelerate colonic transport, soften stool, and increase defecation frequency in a dose-dependent manner (14, 15), which is highly consistent with the clinical characterization of IBS-D. Takeda G-protein-coupled receptor 5 (TGR5), also known as G-protein-coupled bile acid receptor 1, is a BA-responsive receptor with high expression in the intestine and can be activated by CDCA (7). The latest research shows that the expression of TGR5 protein in the rectosigmoid mucosa of IBS-D patients is significantly higher than that of normal people and is positively correlated with clinical abdominal pain and diarrhea symptoms (12).

Although the alteration of some fecal microbiota and BAs has been also reported in IBS-D patients, the role and mechanism of GM and BA in the pathogenesis of IBS-D are largely unknown. In this study, by performing BA-related metabolomic and metagenomic analyses in an IBS-D cohort, the interaction among *Bacteroides ovatus*, bile salt hydrolase (BSH) gene, and BAs was considered to be closely involved with IBS-D patients. Further, through a microbiota-humanized IBS-D rat experiment, we also confirmed that the imbalanced GM–BA–TGR5 axis could promote colonic mucosal barrier dysfunction and enhance visceral hypersensitivity (VH) in IBS-D.

## MATERIALS AND METHODS

### Patients and healthy volunteers

All 25 IBS-D patients were recruited from the Second Clinical College of Guangzhou University of Chinese Medicine from June 2020 to November 2022, and 15 healthy controls (HCs) matched with the IBS-D patients’ age, gender, and body mass index were recruited during the same time. Informed consent was obtained from all eligible participants. Details of the inclusion criteria and exclusion criteria are described in the supplemental material.

### Sample size calculation

Sample size calculation is based on the proportion of conjugated BA in the feces of IBS-D patients and healthy controls in a previous study (16) (IBS-D 3.04 ± 0.77 vs HC 2.35 ± 0.4). It was calculated using the clinical sample size calculation formula, of which the two-tailed significance level was 0.05, the test efficiency (1 − β) was set as 90%, the ratio of the number of cases in the healthy controls to the number of IBS-D patients was 1:2 (*k* = 0.5), and the loss of follow-up rate was considered as 10%. It is calculated that at least 13 healthy subjects and 24 IBS-D patients should be included. The specific calculation formula is as follows:


n=Uα2+Uβ21+1kσ2δ2

α = 0.05, type I error, Uα2 = 1.960; β = 0.1, type II error, *U*_β_ = 1.2816.

κ (ratio of cases in IBS-D group and healthy controls) = 0.5.

δ (the difference in average BA levels between the two groups of people cited in the references) = 0.69.

$\sigma^{2}$ = (κSe^2^ + Sc^2^)/(1 + κ) = 0.3043, Se, the standard deviation of the IBS-D group; Sc, the standard deviation of the HC group.

### Details of the total score of IBS symptom severity scale

This scale evaluates the IBS-D patients’ condition from five dimensions: degree of abdominal pain, frequency of abdominal pain, degree of abdominal distension, dissatisfaction with bowel habits, and quality of life. Each dimension has a score of 0–100 points, with a total score of 500 points. The condition was divided into four levels based on the total score: health level: 0–75 points; mild: 75–174 points; moderate: 175–299 points; and severity: 300–500 points.

### Details of the Bristol fecal traits scale efficacy standard

The Bristol fecal traits scale efficacy standard is as follows: the scores in Table S1 correspond to the scores, type I corresponds to 1 point, type II corresponds to 2 points, and so on.

### Details of IBS-Specific Quality of Life scores

The IBS-Specific Quality of Life (IBS-QOL) questionnaire contains 34 items in total, with 1–5 points for each item and a total score of 170 points. The scores are standardized and inversely scored [final score = 100 − (actual score − theoretical lowest score)/theoretical score range × 100].

### Metagenomic sequencing and analysis

A cetyltrimethylammonium bromide kit was used to extract the micro-DNA of fecal bacteria. After passing the identification and completing the construction of the library, Qubit 2.0, Agilent 2100, and Q-PCR methods were used to dilute the library. The Illumina PE150 (2 × 150) high-throughput sequencing platform was used for sequencing. KneadData software was used to perform quality control (based on Trimmatic) and de-hosting (based on Bowtie2) on the original sequencing data. FastQC was used to detect the rationality and effect of quality control before and after KneadData. Kraken2 was used to identify species in fecal samples and add notes, and then, Bracken was used to predict the actual relative abundance of species in the samples.

### Targeted profiling of bile acids based on UHPLC-MS/MS analysis

Ultra-high-performance liquid chromatography coupled to tandem mass spectrometry (UHPLC-MS/MS) analyses were performed using a Vanquish UHPLC system (Thermo Fisher, Germany) coupled with an Orbitrap Q Exactive HF mass spectrometer. Samples were injected onto a Hypersil Gold column (100 × 2.1 mm, 1.9 µm) using a 17-min linear gradient at a flow rate of 0.2 mL/min. The eluents for the positive polarity mode were eluent A (0.1% FA in water) and eluent B (methanol). The eluents for the negative polarity mode were eluent A (5 mM ammonium acetate, pH 9.0) and eluent B (methanol). The solvent gradient was set as follows: 2% B, 1.5 min; 2%–100% B, 12.0 min; 100% B, 14.0 min; 100%–2% B, 14.1 min; 2% B, 17 min. The Q Exactive HF mass spectrometer was operated in the positive/negative polarity mode with a spray voltage of 3.5 kV, capillary temperature of 320°C, sheath gas flow rate of 35 psi, aux gas flow rate of 10 L/min, S-lens RF level of 60, and aux gas heater temperature of 350°C.

### Animal experiments

#### Rats

Male Wistar rats aged 7 weeks (200 ± 20 g) were provided by the Experimental Animal Center of Southern Medical University and were raised in the Experimental Animal Center of the Second Clinical College of Guangzhou University of Chinese Medicine. The rats were raised in an independently ventilated cage. The temperature of the animal-raising environment was 22 ± 1°C, the humidity was 50% ± 10%, the light and dark cycle was 12 hours, and sufficient food and water were provided. The experiment began after all animals were adaptively raised for 1 week. After confirming the success of the pseudo-germ-free model, the rats were randomly divided into three groups (*n* = 9 per group), including the normal control group (NC), IBS-D group, and TGR5 group (TRIA).

In the CDCA experiment, conventional male Wistar rats aged 7 weeks (200 ± 20 g) were randomly divided into two groups (*n* = 5 per group), the CDCA group (CDCA) was administered gavage CDCA for 4 weeks at a dose of 160 mg/kg (15), and the NC group (NC) was given an equal dose of physiological saline. Other feeding conditions are consistent with the above.

Finally, the blood, colon, and spinal cord of rats were obtained under anesthesia with isoflurane gas inhalation, and rats were sacrificed by cervical dislocation.

### Transplantation of human fecal microbiota into pseudo-germ-free rats

After confirming the success of the pseudo-germ-free model (the detailed methods were described in the supplemental material), all rats rested for 48 hours and were provided sterilized water for drinking. Then, the three groups of rats underwent oral administration with 2 mL of microbiota suspension for 21 days: (i) NC group: transplantation of fecal microbiota from nine randomly selected HC donors, (ii) IBS-D group: transplantation of fecal microbiota from nine randomly selected IBS-D patients, and (iii) TGR5 group: transplantation of fecal microbiota from nine randomly selected IBS-D patients, and TGR5 inhibitor (triamterene, 60 mg/kg) (17, 18) was administered by gavage 2 hours before transplantation. The other two groups were given equal doses of germ-free normal saline by gavage. TGR5 inhibitor was prepared with 5% gum arabic as a suspension aid.

### Detection of gut microbiota of rats by 16S rRNA

Microbial DNA was extracted from fecal samples using the Fast DNA Spin Kit for Soil DNA (MP Biomedicals, Santa Ana, CA, USA) according to the manufacturer’s protocols. The final DNA concentration and purification were determined using a NanoDrop 2000 UV–vis spectrophotometer (Thermo Scientific, Wilmington, NC, USA), and DNA quality was checked by 1% agarose gel electrophoresis. Illumina guidelines were selected to prepare a library of the 16S metagenomic sequencing. QuantiFluor-ST (Promega, USA) was used to quantify according to the manufacturer’s protocol. Sequencing was performed on the Illumina MiSeq PE 300 platform.

Details of the establishment of pseudo-germ-free rats, abdominal withdrawal reflex scale scores, moisture content of the feces, sugar water consumption, open field test, electromyogram recordings of responses to colorectal distension, small intestine propulsion rate, immunohistochemistry, immunofluorescence staining, and enzyme-linked immunosorbent assay (ELISA) are described in the supplemental material.

### Statistical analysis

These metabolites were annotated using the Kyoto Encyclopedia of Genes and Genomes (KEGG) database (https://www.genome.jp/kegg/pathway.html), HMDB database (https://hmdb.ca/metabolites), and LIPIDMaps database (http://www.lipidmaps.org/). Data normalization, principal component analysis, partial least squares discriminant analysis, orthogonal partial least squares discriminant analysis, random forest, and support vector machine were performed with R package MetaboAnalystR (19). To make the data close to a normal distribution, the Normalization function in MetaboAnalystR package (with arguments MedianNorm, LogNorm, and AutoNorm) was adopted. We applied a univariate analysis (*t*-test) to calculate the statistical significance (*P*-value). The metabolites with value importance in projection >1, *P*-value <0.05, and log2(fold change) >1 were considered to be differential metabolites. For clustering heat maps, the data were normalized as *z*-scores and plotted by the Pheatmap package in R language. A volcano plot was used to filter metabolites of interest based on log2(fold change) and −log10(*P*-value), which was conducted by the ggplot2 package in R language. The metabolites with a *P*-value <0.05 (*t*-test) were used to conduct an over-representation analysis (ORA) enrichment analysis, and the resulting KEGG pathways with *P*-value <0.05 (ORA) were considered as statistically significant enrichment.

SPSS 23.0 was used for other statistical analyses, and GraphPad Prism 7.00 software was used for drawing. Enumeration data were tested by the χ^2^ test; measurement data were expressed as the mean ± standard deviation (*x̅* ± *s*) or *M* (*P*25, *P*75). A *t*-test was used for comparison between two groups, and the least significant difference (LSD)*-t*-test was used for comparison among multiple groups with normal distribution and homogeneity of variance. Welch’s *t*-test was used for comparison between two groups, and the Dunnet *T*3 test was used for comparison among multiple groups with normal distribution but unequal variance. The Mann–Whitney *U* rank sum test was used for data that did not conform to normal distribution between two groups; the Kruskal–Wallis *H* rank sum test was used for data that did not conform to normal distribution among three or more groups. *P* < 0.05 was statistically significant.

## RESULTS

### Demographics and clinical characteristics

As shown in Table 1, 25 IBS-D patients and 15 HCs participated in the study. There was no abnormality and no obvious difference in the blood routine test, liver function alanine transaminase (ALT), aspartate aminotransferase (AST), renal function creatinine (Cr), blood urea nitrogen (BUN), and serum total BA of subjects between the two groups. However, the IBS-D cohort displayed a significant increase in defecation frequency.

**TABLE 1:** Demographics and clinical characteristics

Parameter	Healthy control (HC)	IBS-D group
*N*	15	25
(Men:women）	7:8	16:9
Age (years)	27.00 (25.00, 28.00)	25.00 (22.00, 31.50)
BMI (kg/m^2^)	22.10 ± 2.63	21.08 ± 2.91
Stool frequency (n/d)	1.00 (1.00, 1.00)	3.00 (3.00, 4.00)^*a*^
WBC (10^9^/L)	5.78 ± 1.04	5.73 ± 1.22
Hb (g/L)	145.67 ± 17.11	147.96 ± 17.01
ALT (U/L)	13.00 (10.00, 19.00)	8.50 (8.00, 15.00)
AST (U/L)	17.00 (10.00, 19.00)	17.00 (15.00, 21.00)
Serum total BA (µmol/L)	3.80 (2.10, 5.40)	3.00 (2.00, 4.45)
BUN (U/L)	4.24 ± 0.82	3.96 ± 0.64
Cr (µmol/L)	71.60 ± 15.13	70.60 ± 16.39
IBS-SSS score	NA	198.67 ± 3.56
IBS-QOL score	NA	69.67 ± 1.38
Bristol stool form scale score	NA	6.00 (5.25, 7.00)

^*a*^ *P* < 0.001, compared with the HC group. Gender comparison between two groups uses the χ^2^ test. Data that conform to the normal distribution are represented by χ̄̅ ± ѕ.

For data that conform to the normal distribution and homogeneity of variances, the *t*-test is used for comparison between the two groups. For data that conform to the normal distribution but have uneven variances, Welch’s *t*-test is used for comparison between the two groups. For data that do not conform to the normal distribution, the Mann–Whitney *U* rank sum test is used for the comparison between the two groups.

### Characteristics of the gut microbiota in IBS-D patients

**Fig 1:**
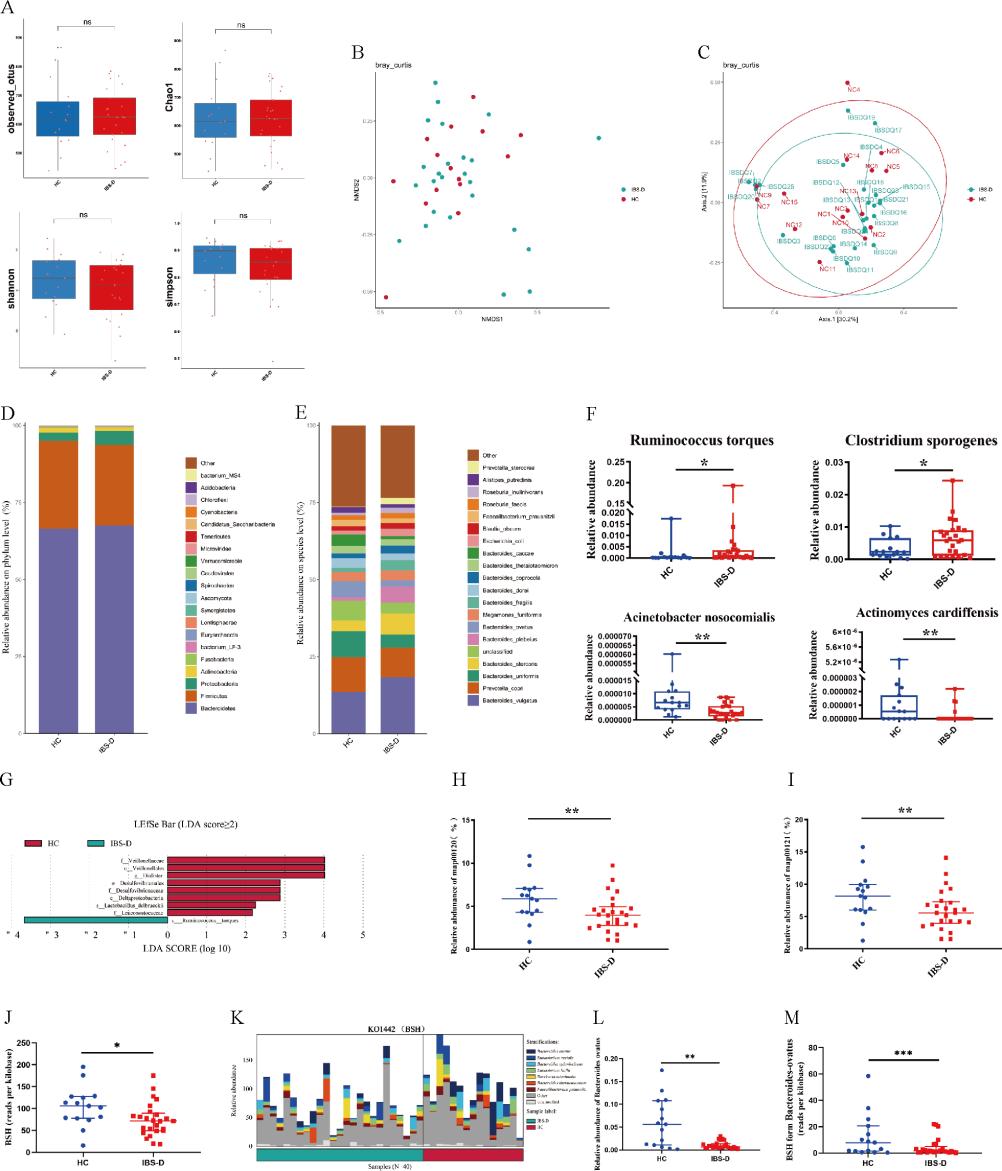
Profiling of the gut microbiota in IBS-D and HC groups. (**A**) Alpha diversity was evaluated by observed operational taxonomic units (OTUs), Chao1, Shannon, and Simpson index. (**B and C**) Beta diversity was calculated by PCoA and NMDS based on the Bray–Curtis distance. (**D and E**) Relative abundances of gut microbiota at phylum and species level. (**F**) The two species ranked in order with the most increased relative abundance in IBS-D and HC groups, respectively. (**G**) Biomarkers of discriminative bacteria (from phylum to species) in the IBS-D and HC groups identified by linear discriminant analysis effect size (LEfSe) analysis. (**H and I**) KEGG annotation of key altered BA metabolic pathways in IBS-D and HC groups. (**J**) Relative abundances of BSH gene in IBS-D and HC groups. (**K**) Species origin of BSH gene in IBS-D and HC groups. (**L**) Relative abundances of *Bacteroides ovatus* in IBS-D and HC groups. (**M**) BSH gene composition from *Bacteroides ovatus* in IBS-D and HC groups. Data are presented as medians with interquartile ranges. The Dunnet *T*3 test is used for comparison among multiple groups with normal distribution but unequal variance. Significant differences are represented by ^***^P < 0.001, compared to the HC group.

Compared to the HC group, fecal microbial communities exhibited a rising trend of observed and Chao1 index in the IBS-D group but a downward trend of Simpson and Shannon index in the IBS-D group (Fig. 1A). Moreover, the results of the nonmetric multidimensional scaling (NMDS) (Fig. 1B) and principal coordinate analysis (PCoA) plot (Fig. 1C) based on Bray–Curtis distance also showed no evident differences in the global microbiota structure between IBS-D patients and HCs. These results suggested the unchanged microbial richness within all included subjects but a larger instability of the enteric ecosystem in the IBS-D subgroup.

Further analysis showed that a different microbial profile was found in IBS-D patients in comparison with HC subjects at different taxonomic levels. At the phyla level, *Firmicutes* and *Bacteroidetes* accounted for more than 90% of both HCs and IBS-D populations, and we did not find any significant differences (Fig. 1D). At the species level, 91 species were significantly less abundant in the fecal microbiota of IBS-D patients, including 52 species belonging to *Firmicutes*, 18 species belonging to *Bacteroidetes*, and 21 other reduced species belonging to *Proteobacteria*, *Actinobacteria*, *Synergistetes,* and *Tenericutes*, respectively (Fig. 1E). Moreover, the two species ranked in order with more reduced relative abundance in IBS-D patients were *Acinetobacter_nosocomialis* and *Actinomyces_cardiffensis*, and the two species ranked in order with the most increased relative abundance in IBS-D patients were *Ruminococcus torques* and *Clostridium_sporogenes* (Fig. 1F). Subsequently, LEfSe analysis indicated that *Lactobacillus delbrueckii* and *Ruminococcus torques* may be the most important species to distinguish the two groups of people (Fig. 1G).

KEGG analysis indicated that BA metabolism was one of the key metabolic pathways (primary BA biosynthesis [map00120] and secondary BA biosynthesis [map00121]) affected by the GM changes found in IBS-D (Fig. 1H and I). Moreover, the alteration in the bacterial composition of the IBS-D group was also associated with variation in BA-transforming genomes. A significant reduction in the abundance of BSH genes was observed in IBS-D compared with HCs (Fig. 1J). Meanwhile, KO1442 (BSH) gene mainly came from *Bacteroides ovatus* (Fig. 1K). Interestingly, both the abundance of *Bacteroides ovatus* and BSH genes were observed to decrease significantly in IBS-D compared with HCs (Fig. 1L and M). These data indicated that GM was significantly changed in IBS-D, which in turn influenced the metabolism of BAs in these hosts, especially the interaction among the *Bacteroides ovatus*, KO1442 (BSH) gene, and BAs.

### Fecal BA pool composition in IBS-D patients

**Fig 2:**
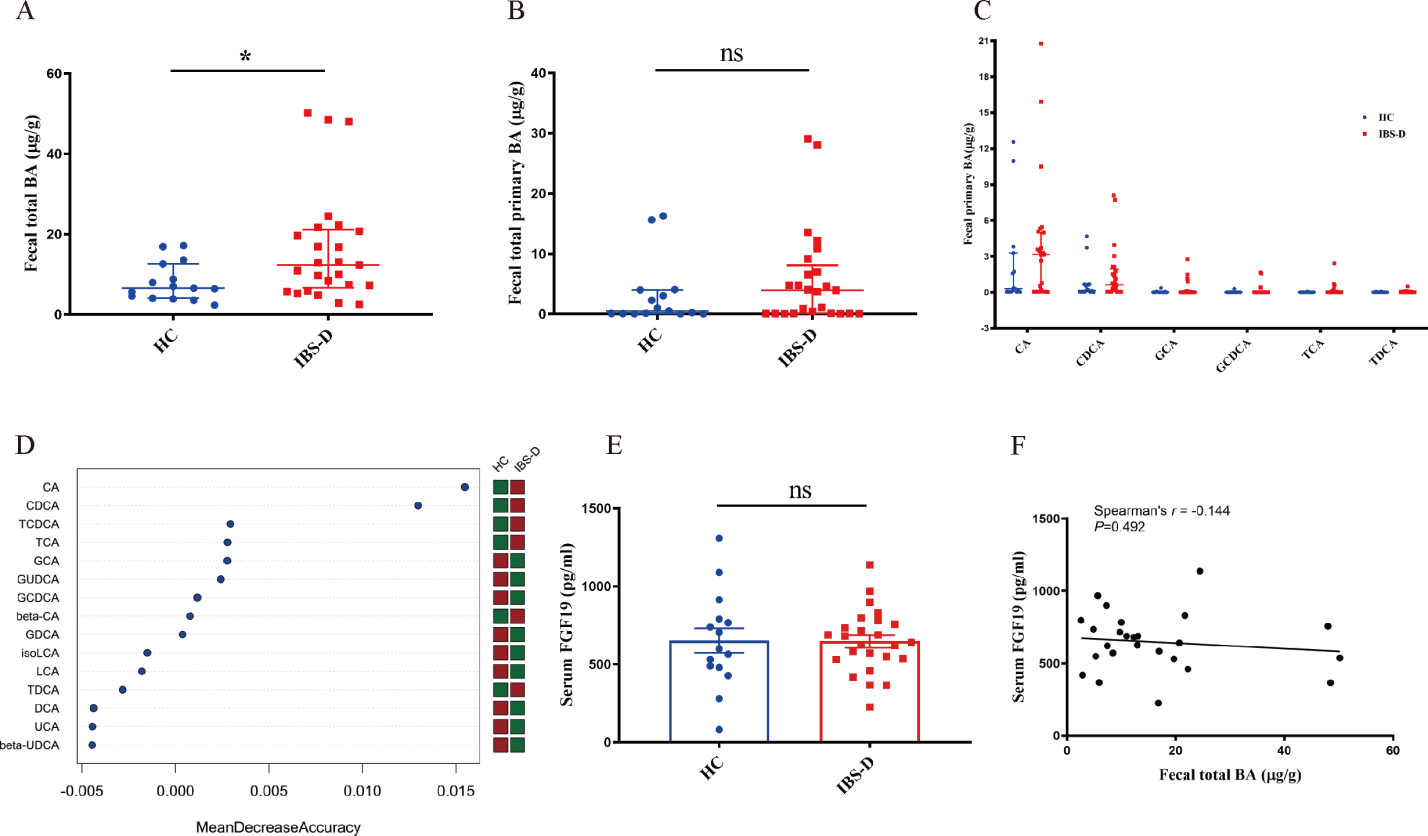
Profiling of the fecal BA in HC group and IBS-D patients. (**A and B**) Levels of fecal total BAs and fecal total primary BAs in two groups. (**C**) Levels of CA, CDCA, glycocholic acid, glycochenodeoxycholic acid, taurocholic acid, and taurochenodeoxycholic acid in two groups. (**D**) Random forest importance plot for BAs in two groups. (**E**) Levels of serum FGF19 in HC and IBS-D group. (**F**) Correlations between fecal BAs and serum FGF19. Data are presented as medians with interquartile ranges. The Mann–Whitney *U* rank sum test is used for comparison between the two groups that the data do not conform to the normal distribution. Welch’s *t*-test is used for comparison between two groups that the data conform to normal distribution but unequal variance. Significant differences are represented by ^*^P < 0.05, compared to the IBS-D group.

As shown in Fig. 2A, the total BAs were significantly elevated in IBS-D patients compared to HCs. Total primary BAs showed an increased trend in IBS-D patients although not significant when compared with HCs (Fig. 2B). Furthermore, the median expression level of primary BAs CDCA and cholic acid (CA) also had an obvious rise in IBS-D patients, but it lost significance after false discovery rate (FDR) correction (Fig. 2C). In addition, it was worth noting that CDCA and CA contributed significantly to group separation based on random forest, which may be the most important BA metabolites in IBS-D (Fig. 2D). The sequencing results of GM and BA metabolism revealed a changed composition of microbiota-derived BAs (e.g., CDCA and CA) in the feces of IBS-D patients, indicating that an abnormality in BA-transforming GM might contribute to the cause of this condition.

To determine the synthesis of BAs in the liver of the two groups, we detected the serum FGF19 expression level of the two groups by ELISA. It showed that there was no significant difference in serum FGF19 expression between the IBS-D patients and HCs (Fig. 2E). The result indicated that the cases included in this study were IBS-D patients rather than BA diarrhea patients. Moreover, we found a weak negative correlation between fecal total bile acid and serum FGF19 in IBS-D patients but no significance (Fig. 2F).

### The influence of humanized FMT on gut microbial composition in rats

**Fig 3:**
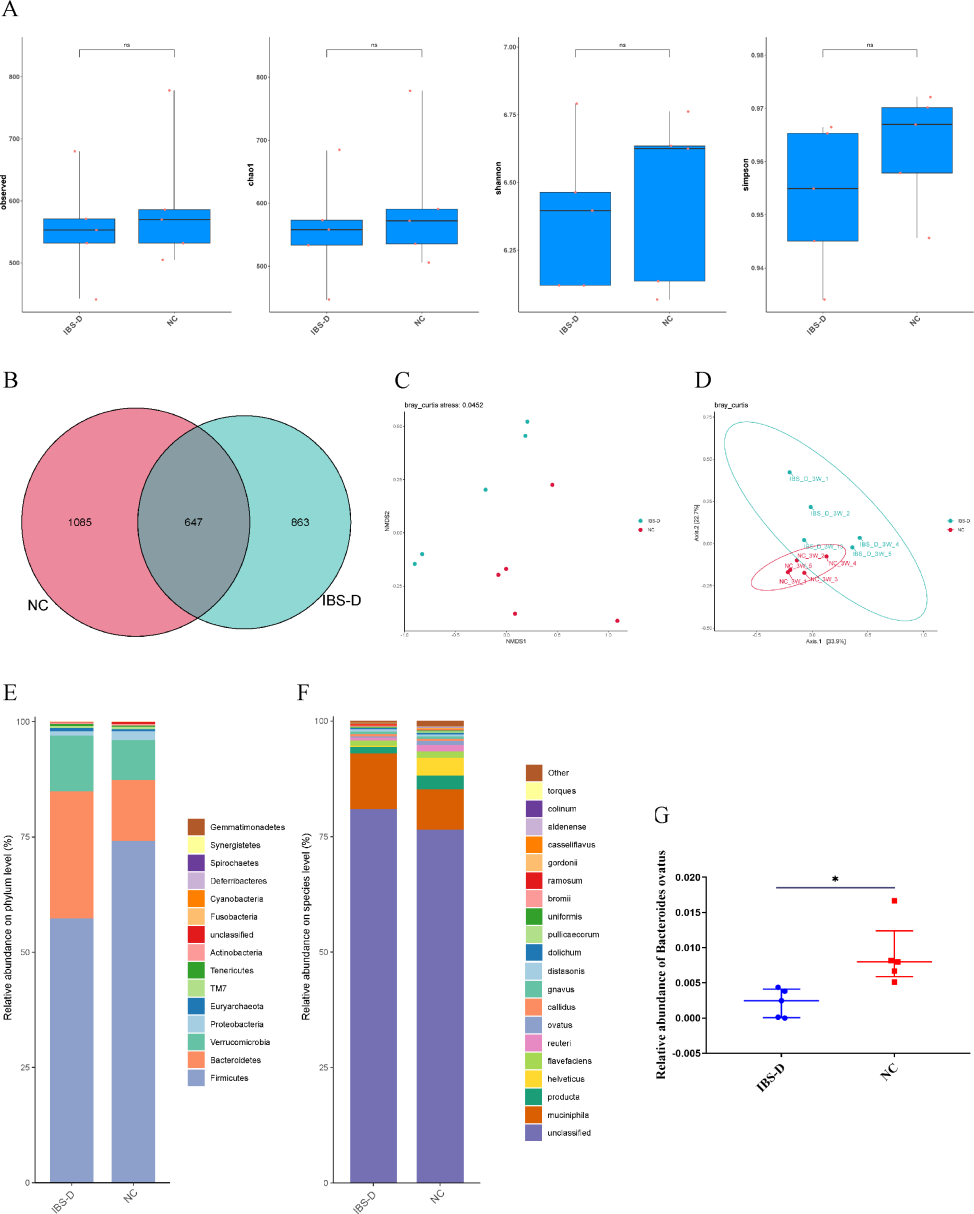
The influence of humanized FMT on gut microbial composition in rats. The influence of humanized FMT on gut microbial composition in rats. (**A**) Alpha diversity was evaluated by observed, Chao1, Shannon, and Simpson index. (**B**) Comparison of OTUs between the NC and IBS-D groups. (**C and D**) Beta diversity was calculated by NMDS and PCoA based on the Bray–Curtis distance. (**E and F**) Relative abundances of gut microbiota at phylum and species level. (**G**) The relative abundance of *Bacteroidetes ovatus* in the NC and IBS-D groups. For data that do not conform to the normal distribution, the Mann–Whitney *U* rank sum test is used for the comparison between the two groups. Significant differences are represented by ^*^P < 0.05. ns, no significance, compared to the IBS-D group.

After antibiotic treatment to deplete the intestinal microbiota for 4 weeks, rats received feces from healthy people and IBS-D patients for 3 weeks. We detected the gut microbiota composition of rats. The observed, Chao1, Simpson, and Shannon index had no difference between the NC and IBS-D groups (Fig. 3A). However, the IBS-D group has 222 fewer OTUs than the NC group (Fig. 3B). Moreover, the results of NMDS (Fig. 3C) and PCoA (Fig. 3D) based on Bray–Curtis distance showed distinctive microbial communities in the fecal microbiota between the NC and IBS-D groups. We further analyzed the differences in gut microbiota composition between the two groups. At the phylum level, the relative abundance of *Firmicutes* was decreased in the IBS-D group, but *Bacteroidetes* was enriched (Fig. 3E). At the species level, *Bacteroidetes ovatus* was significantly diminished in the IBS-D group (Fig. 3F and G), which was consistent with changes in the above human GM. Therefore, the alteration of *Bacteroidetes ovatus* suggested that humanized fecal microbial transplantation (FMT) could re-establish part of the donor microbiota in the gut of pseudo-germ-free rats.

### The influence of GM–BA–TGR5 axis on the phenotype, behaviors, and pathology of IBS-D

**Fig 4:**
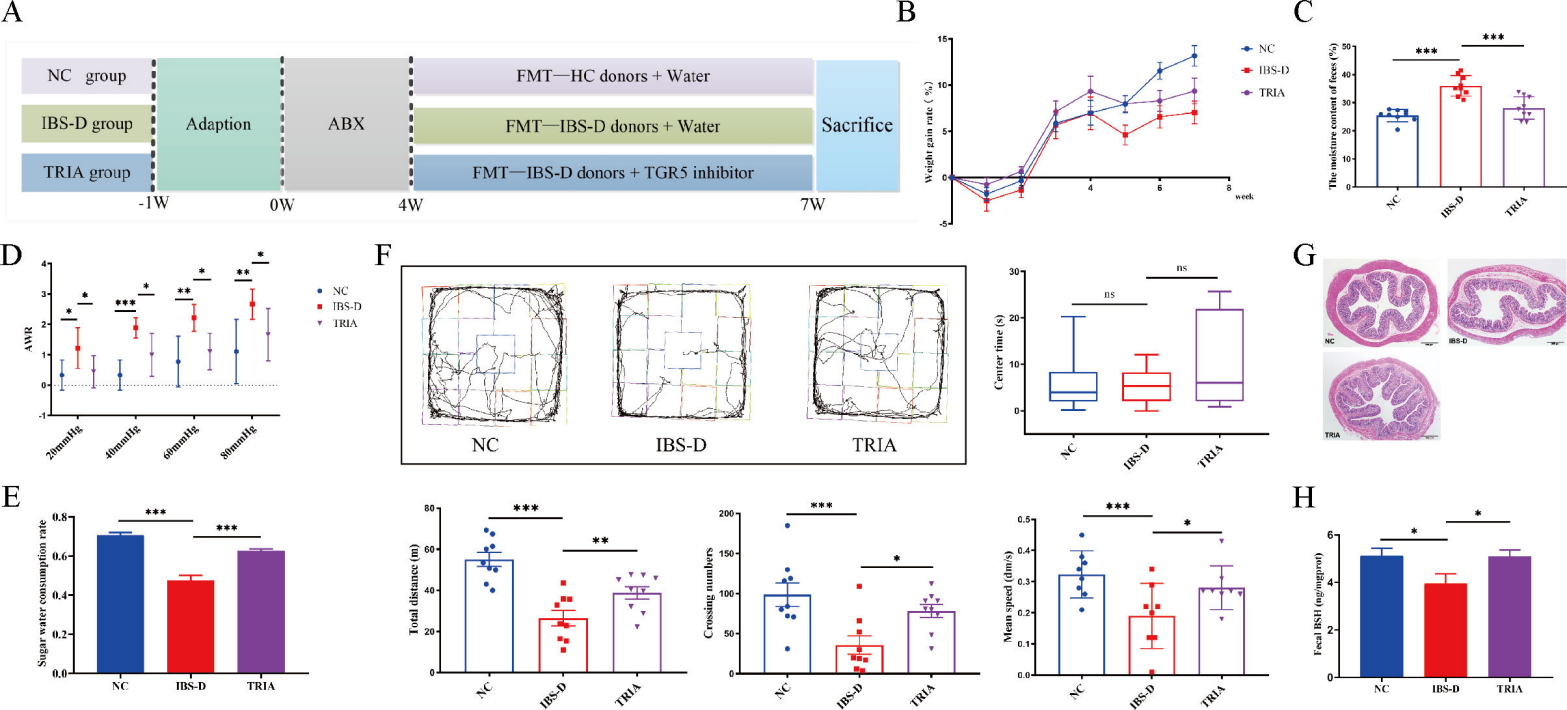
The influence of GM–BA–TGR5 axis on the phenotype, behaviors, and pathology of IBS-D. (**A**) Experimental procedure for FMT in the antibiotic cocktail-induced (ABX-induced) pseudo-germ-free rats (*n* = 9/group). ABXs were given to mice in the drinking water for 4 weeks. Rats that received fecal microbiota of HC donors were grouped as the NC group, rats treated with fecal microbiota from IBS-D patients were classified as the IBS-D group, and rats that received fecal microbiota of IBS-D patients and TGR5 inhibitor were grouped as the TRIA group. (**B**) The 7-day weight gain rate (%) of rats in four groups. (**C**) The moisture content of the feces in each group. (**D**) The AWR score of rats at distension pressures of 20, 40, 60, and 80 mmHg. (**E**) The sugar water consumption rate in four groups. (**F**) Automated tracking in the open field test of each rat, including path map of rats in each group, total distance (cm), crossing numbers, average speed (dm/s), and center time (s). (**G**) H&E stain of colon tissues in different groups (40×). (**H**) The expressions of BSH proteins in feces of four groups. Data are presented as the mean ± SD or medians with interquartile ranges. The LSD-*t*-test is used for comparison among multiple groups with normal distribution and homogeneity of variance. The Kruskal–Wallis *H*rank sum test is used for data that do not conform to normal distribution among three groups. Significant differences are represented by ^*^P < 0.05, ^**^P < 0.01, and ^***^P < 0.001, compared to the IBS-D group.

To investigate the effect of the GM–BA axis on IBS-D, we successfully established and evaluated the pseudo-germ-free rat model (Fig. S1). Then, stools from HCs or IBS-D patients were transplanted into pseudo-germ-free rats by oral gavage (Fig. 4A). Compared with the NC group, the IBS-D group displayed a decrease in the weight gain rate (Fig. 4B), increased moisture content of the feces (Fig. 4C), elevated score of abdominal withdrawal reflex (AWR) (Fig. 4D), and more depression-like behaviors (Fig. 4E and F). Moreover, the hematoxylin and eosin (H&E) staining demonstrated little neutrophil infiltration, decreases in the number of crypts in some mucosal segments, and a slight increase in the distance between the two crypts within tissues from the IBS-D model group (Fig. 4G). These changes could be significantly reversed by the TGR5 inhibitor. Therefore, our results demonstrated that human-associated FMT could successfully induce the IBS-D rat model and upregulated TGR5 involved in the pathogenesis of IBS-D.

To understand whether the influence of GM on the conversion of BAs can be transferred with FMT, we used ELISA to determine the expression of fecal BSH. In the intestine, the BSH enzyme is produced by intestinal bacteria and catalyzes the conversion of primary BA to secondary BA (20). As shown in Fig. 4H, the IBS-D group indicated a significant decrease in fecal BSH when compared to the NC group, which was consistent with the result that the relative abundance of the BSH gene in IBS-D patients was significantly lower than that in healthy subjects in clinical observations, but the TRIA group had an obvious increase. These results further suggested that the dysfunctional GM of IBS-D patients could still inhibit the BA metabolism of the recipients after FMT. Taken together, our findings indicated that the GM–BA–TGR5 axis could play a vital role in IBS-D.

### The influence of GM–BA–TGR5 axis on colonic mucosal barrier function and VH of IBS-D

**Fig 5:**
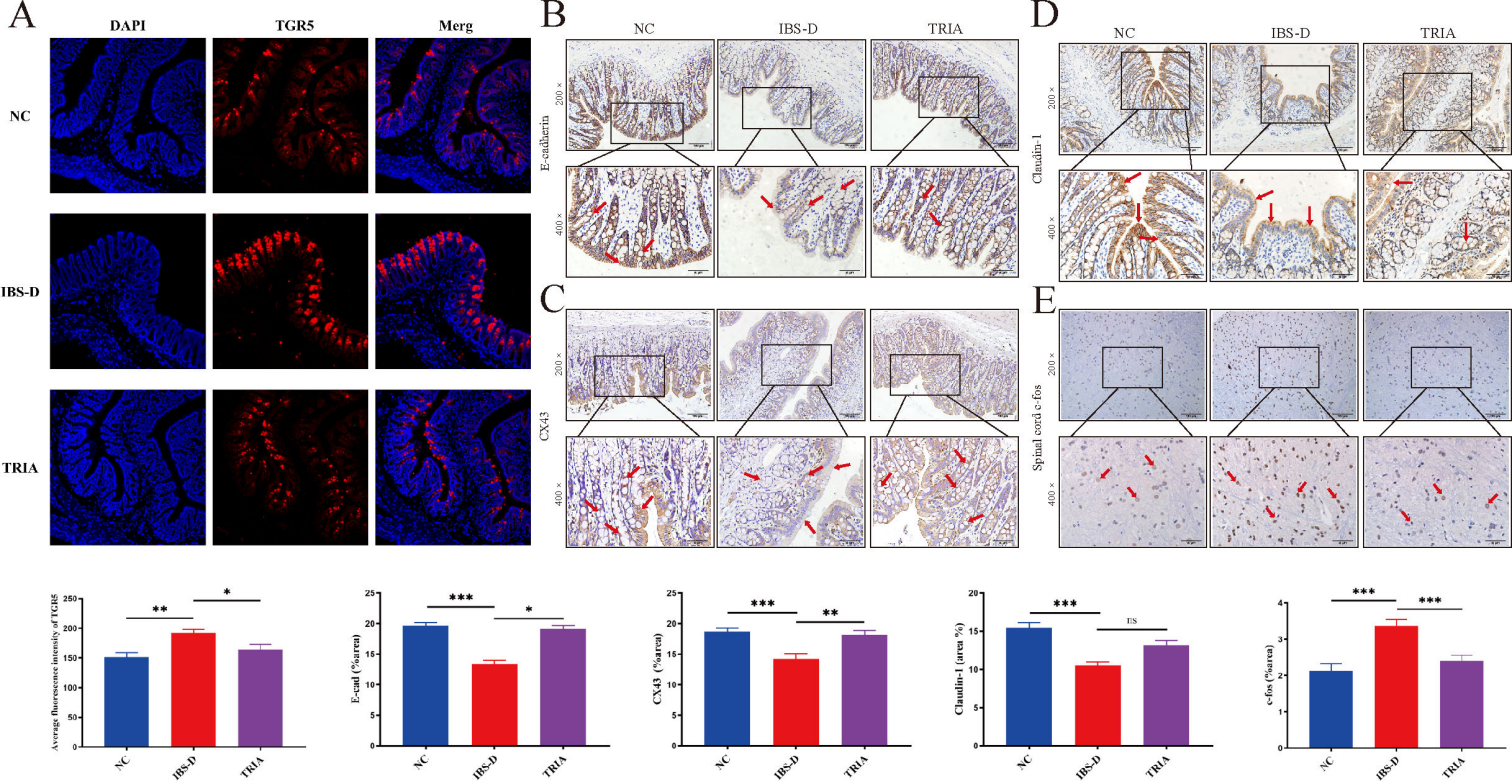
The influence of GM–BA–TGR5 axis on colonic mucosal barrier and VH of IBS-D. (**A**) Immunofluorescence images of TGR5 in each group, scale bar for the 200× magnification: 50 µm. (**B through D**) Representative immunohistochemistry images of junction proteins (E-cadherin, CX43, and Claudin-1) in the three groups. (**E**) Representative immunohistochemistry images of c-fos proteins in the spinal cord. Scale bar for the 200× magnification: 100 µm; for the 400× magnification: 50 µm. Data are presented as the mean ± SD. The LSD-*t*-test is used for comparison among multiple groups with normal distribution and homogeneity of variance. Significant differences are represented by ^*^P < 0.05, ^**^P < 0.01, and ^***^P < 0.001, compared to the IBS-D group.

To further explore how the GM–BA–TGR5 axis plays a regulatory role in IBS-D, we detected alterations in TGR5-relevant signal pathways. Compared to the NC group, immunofluorescence analysis showed that the IBS-D group had a significant increase in the expressions of TGR5, which was mainly expressed on the enterochromaffin cell membrane of the colonic crypt and mucosal epithelium, showing a mass of irregular agglomerate fluorescence. However, it could be downregulated by TGR5 inhibitor (Fig. 5A). Moreover, immunohistochemistry analysis suggested that the mucosal barrier protein E-cadherin, CX43, and Claudin-1 in the colon were mainly expressed on the membrane, and the expression of them had an obvious decrease in the IBS-D group when compared with the NC group. Meanwhile, these mucosal barrier proteins were upregulated by TGR5 inhibitor treatment (Fig. 5B through D). Collectively, our data demonstrated that the imbalanced GM–BA–TGR5 axis promotes dysfunction of the colonic mucosal barrier in IBS-D.

Another important clinical manifestation of IBS-D is abdominal pain or abdominal discomfort, which is mainly involved in VH. Therefore, we continued testing changes in TGR5-relevant pathways about VH. As shown in Fig. 5E, immunohistochemistry showed that c-fos-positive cells were mainly expressed in the cytoplasm of neurons in the spinal dorsal horn of rats in each group, appearing brown. Moreover, the IBS-D group had a higher level of c-fos in the spinal cord, while the TGR5 inhibitor suppresses its upregulation. Overall, our findings demonstrated that colonic barrier protein and c-fos protein in the spinal cord were the important targets of the GM–BA–TGR5 axis, and their regulation may account for the effect of the imbalanced GM–BA–TGR5 axis in the promotion of the diarrhea and abdominal pain in IBS-D.

### CDCA induces VH through upregulating the TGR5 receptor in the colon and terminal ileum

The above clinical and animal experimental studies proved that the main performance of the BAs in the GM–BA–TGR5 axis was the increase of CDCA (Fig. 2D). Previous studies observed that CDCA was significantly increased in IBS-D patients (4, 21), but few researches had been carried out on its specific mechanism. To further explore whether CDCA could induce diarrhea and VH through the TGR5 receptor, oral gavage with CDCA for 4 weeks was conducted for rats.

**Fig 6:**
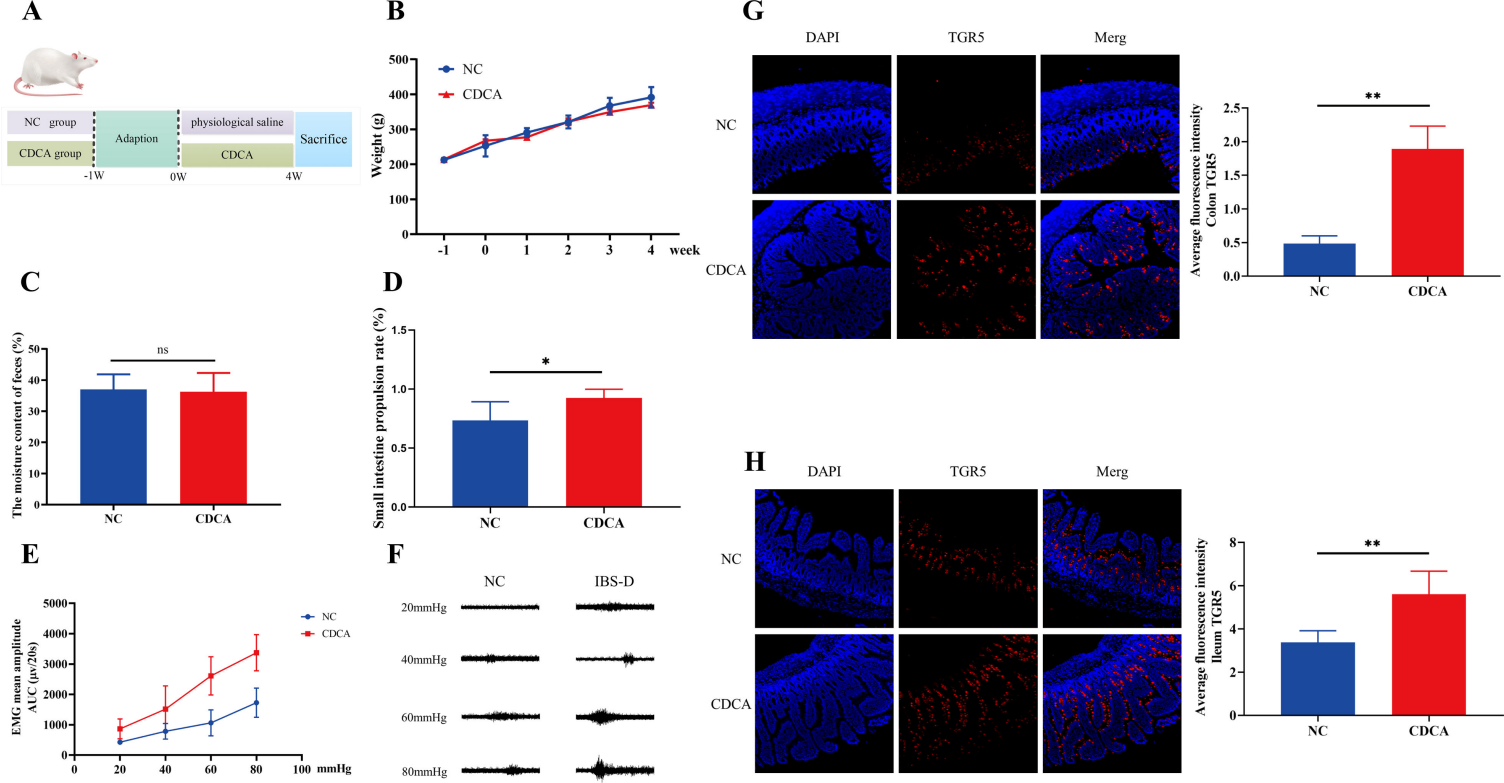
CDCA induces VH through upregulating the TGR5 receptor in the colon and terminal ileum. (**A**) Protocol diagram of the time course involved in the experimental procedures of two groups. (**B**) The weight changes of rats in two groups. (**C**) The moisture content of the feces in each group. (**D**) The small intestine propulsion rate (%) in two groups. (**E**) Mean amplitudes of abdominal muscle contractions are expressed as the area under the curve after baseline subtraction. (**F**) Representative electromyogram recordings of responses in rats. (**G and H**) The expression level of TGR5 in the colon and ileum. Data are presented as the mean ± SD. A *t*-test is used for comparison between two groups with normal distribution and homogeneity of variance. Significant differences are represented by ^*^P < 0.05 and ^**^P < 0.01, compared to the CDCA group.

Notably, the CDCA group showed no significant difference in body weight (Fig. 6B) and the moisture content of the feces (Fig. 6C) but had an obvious increase in the score of electromyography (Fig. 6Eand F) compared with the NC group, which indicated the excellent VH-inducing effect of CDCA. Moreover, we found that CDCA could increase the small intestine propulsion rate but not cause diarrhea (Fig. 6D). Immunofluorescence analysis indicated that TGR5 protein in the colon and terminal ileum had a significant increase in the CDCA group compared with the NC group (Fig. 5G and H). These data indicated that CDCA may induce VH and intestinal motility through the TGR5 receptor in the colon and terminal ileum. Meanwhile, it also suggested that IBS-D may be the common result of the changes of various BAs, not only CDCA.

## DISCUSSION

In the present study, we first revealed the characteristics of an imbalanced GM–BA axis in IBS-D patients and the correlation between GM–BA and IBS-D patients. Next, we explored the underlying mechanism of diarrhea and abdominal pain in IBS-D, which was found to be involved with colonic barrier dysfunction and VH mediated by the GM–BA–TGR5 axis.

Evidence is accumulating that the composition of the GM has alternation in IBS-D (5, 8). At the species level, the proportions of the *Ruminococcus torques* and *Clostridium sporogenes* have been observed to increase in IBS-D patients. The increase in the relative abundance of *Ruminococcus torques* was not only verified in the IBS-D rat model (22) but also reported to be highly positively correlated with the number of enterochromaffin cells, HAM-D scale score, degree of abdominal pain, and frequency of abdominal pain in IBS-D patients (23). Similarly, the phenomenon that *Clostridium sporogenes* is highly enriched in IBS-D patients is also very interesting, because it is found that spore-forming bacteria mainly composed of Clostridia Cluster IV and XIVa can also stimulate enterochromaffin cells in the colon to synthesize 5-HT (24). Ninety-five percent of 5-HT in the human body is stored in enterochromaffin cells and intestinal neurons. Metabolites of GM, such as BAs and short-chain fatty acids, can act on host enterochromaffin cells to, respectively, stimulate the release of TGR5 and tryptophan hydroxylase 1, resulting in the upregulation of the expression of 5-HT (7, 24). Our findings are highly consistent with the reports on the relationship between intestinal 5-HT imbalance and IBS-D. Meanwhile, LEfSe analysis showed that *Ruminococcus torques* may be the potential GM biomarkers for IBS-D patients, which confirmed the vital role of *Ruminococcus torques* in IBS-D once again and would be the focus of later research.

GM participates in host metabolism by interacting with host signaling pathways. KEGG analysis showed that primary and secondary BA metabolic pathways had an obvious decrease in IBS-D patients compared with HCs; moreover, the abundance of KO1442 (BSH) gene that regulated the conversion of primary BA into secondary BA was also significantly reduced. Notedly, our qualitative and quantitative analyses of BA species indicated that total BA and total primary BA were raised in IBS-D patients compared with HCs, which verified the reliability of the KEGG analysis. Moreover, we found that the median expression level of CDCA also had obviously raised in IBS-D patients, though it lost significance after FDR correction. These results were in agreement with recent studies by Zhao et al. and Wei et al. (5, 12). Meanwhile, our results also confirmed that CDCA may induce VH and increased intestinal motility through the TGR5 receptor in the colon and ileum. HUMAnN2 software analyzed the origin of microbial species with the function of the KO1442 (BSH) gene showing that *Bacteroides ovatus*, whose relative abundance was reduced, may be one of the important bacteria that affect this function. It indicated that reduced *Bacteroides ovatus* may influence the expression of the KO1442 (BSH) gene to affect the metabolism of primary BAs in these hosts.

Microbes are the main component transferred in FMT (25), and FMT causes changes in intestinal microbial composition that can last for several months (26). Interestingly, we found that the relative abundance of *Bacteroides ovatus* decreased in both IBS-D patients and rats with FMT derived from IBS-D patients. On the one hand, we confirmed that humanized FMT could re-establish part of the donor microbiota in the gut of pseudo-germ-free rats. On the other hand, we also demonstrated the reliability of KEGG prediction results about *Bacteroides ovatus* and BSH in metagenomes. Moreover, by detecting fecal BSH in the humanized rat model, our results further found that GM from IBS-D patients could still make a suppressive effect on the BA metabolism of recipients after FMT. Therefore, it also raises the possibility that gut microbiota-oriented therapeutic strategies may be beneficial in treating not only intestinal symptoms but also the metabolism of IBS (27).

TGR5, as an important receptor of BA, has been demonstrated to participate in the regulation of colonic function (7). Recently, elevated TGR5 in rectosigmoid mucosa was observed in IBS-D patients (12). Moreover, animal research found that colonic transit was 2.2-fold faster and defecation frequency was increased 1.4-fold in TGR5^+/+^ mice than the TGR5-wt mice (28). Notably, the activation of TGR5 was involved in the regulation of cell proliferation in digestive organs and reduced intestinal barrier function (29, 30). Claudin-1, E-cadherin, and CX43 are the representative protein of tight junctions, adherens junctions, and gap junctions, respectively. Our results showed the TGR5 inhibitor could downregulate TGR5 protein and upregulate the expression of Claudin-1, E-cadherin, and CX43 proteins. Taken together, it is reasonable to speculate that the imbalanced GM–BA–TGR5 axis is involved in the intestinal barrier dysfunction of IBS-D.

VH is another vital pathogenesis of IBS-D, and its external manifestation is abdominal pain. Recent studies found that intracolonic administration of TGR5 agonists in mice induced increased VH, which was connected with the increased secretion of 5-HT (31). Further research shows that increased 5-HT could promote the upregulation of c-fos in the spinal cord and brain, which is a probable mechanism of IBS-D (32). c-fos is a recognized marker of neural activation, which is mainly expressed in the nucleus of neurons related to pain transmission (33). So, we evaluated the expression and location of c-fos in the spinal cord by using immunohistochemistry. Our findings indicated that the distribution of c-fos was significantly increased in the IBS-D group, but this phenomenon could be reversed in the TRIA group. Overall, it is also reasonable to speculate that the imbalanced GM–BA–TGR5 axis promotes the VH of IBS-D.

There were several limitations in this preliminary study. Firstly, some BAs in IBS-D patients had shown an increasing or decreasing trend, but there was no statistical significance maybe due to the small sample size. Secondly, the gut microbiota-interfered factor that cannot be ignored in studies of human beings is dietary habits. A standardized diet for subjects is not provided during the study period, and the subjects’ short-term alternations of diet could rapidly disturb the GM.

**Fig 7:**
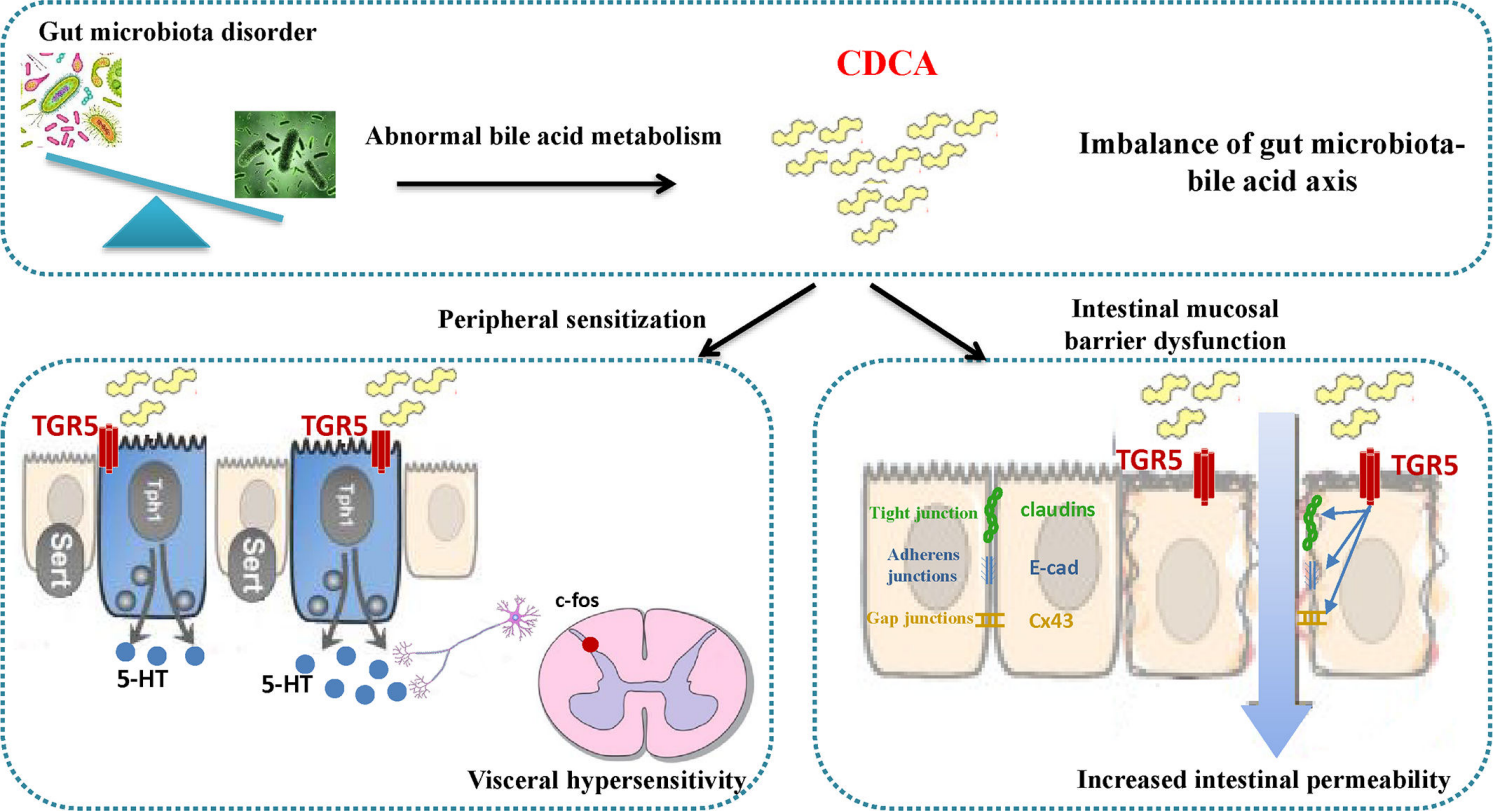
The imbalanced GM–BA–TGR5 axis may promote colonic mucosal barrier dysfunction and enhances VH in IBS-D.

In conclusion, the combination of human omics analysis and mechanistic experiments revealed that the composition of the fecal BA pool in IBS-D patients was characterized by increased total and primary BA, which may be induced by dysbiosis in IBS-D, especially the reduction of GM associated with BSH activity such as *Bacteroides ovatus*. Although CDCA may induce VH and intestinal motility through the TGR5 receptor in the colon and terminal ileum, it was not the only abnormal bile acid factor that causes IBS-D. Moreover, our study showed that the imbalanced GM–BA–TGR5 axis may promote colonic mucosal barrier dysfunction and enhance VH in IBS-D (Fig. 7). However, we measured the expression level of BSH instead of BAs in rats, resulting in the inability to visually determine changes in BAs. Thus, the conclusions drawn in this study need to be interpreted carefully, and further improvements need to be made in subsequent experiments.

## Data Availability

Data from the Illumina sequences analyzed for this project are available at the NCBI Sequence Read Archive (SRA) as BioProject accession number PRJNA1000586 (biosamples SAMN 36771489 through SAMN 36771528; SRA experiments SRR 25515982 through SRR 25515943).

## SUPPLEMENTAL MATERIAL

Figure S1 msystems.01299-23-s0001.tif Assessment of the antibiotic cocktail-induced pseudo-germ-free rat.

Supplemental text msystems.01299-23-s0002.DOCX Methodology of clinical section and animal experiments; legend for Fig. S1.

Table S1 msystems.01299-23-s0003.DOCX The Bristol stool form scale.

ASM does not own the copyrights to Supplemental Material that may be linked to, or accessed through, an article. The authors have granted ASM a non-exclusive, world-wide license to publish the Supplemental Material files. Please contact the corresponding author directly for reuse.
