# Supplementary figures and images for: The function of the gut microbiota–bile acid–TGR5 axis in diarrhea-predominant irritable bowel syndrome
Source: mSystems. 2024 Feb 8;9(3):e01299-23. doi: 10.1128/msystems.01299-23 (PMC10949424; doi:10.1128/msystems.01299-23)

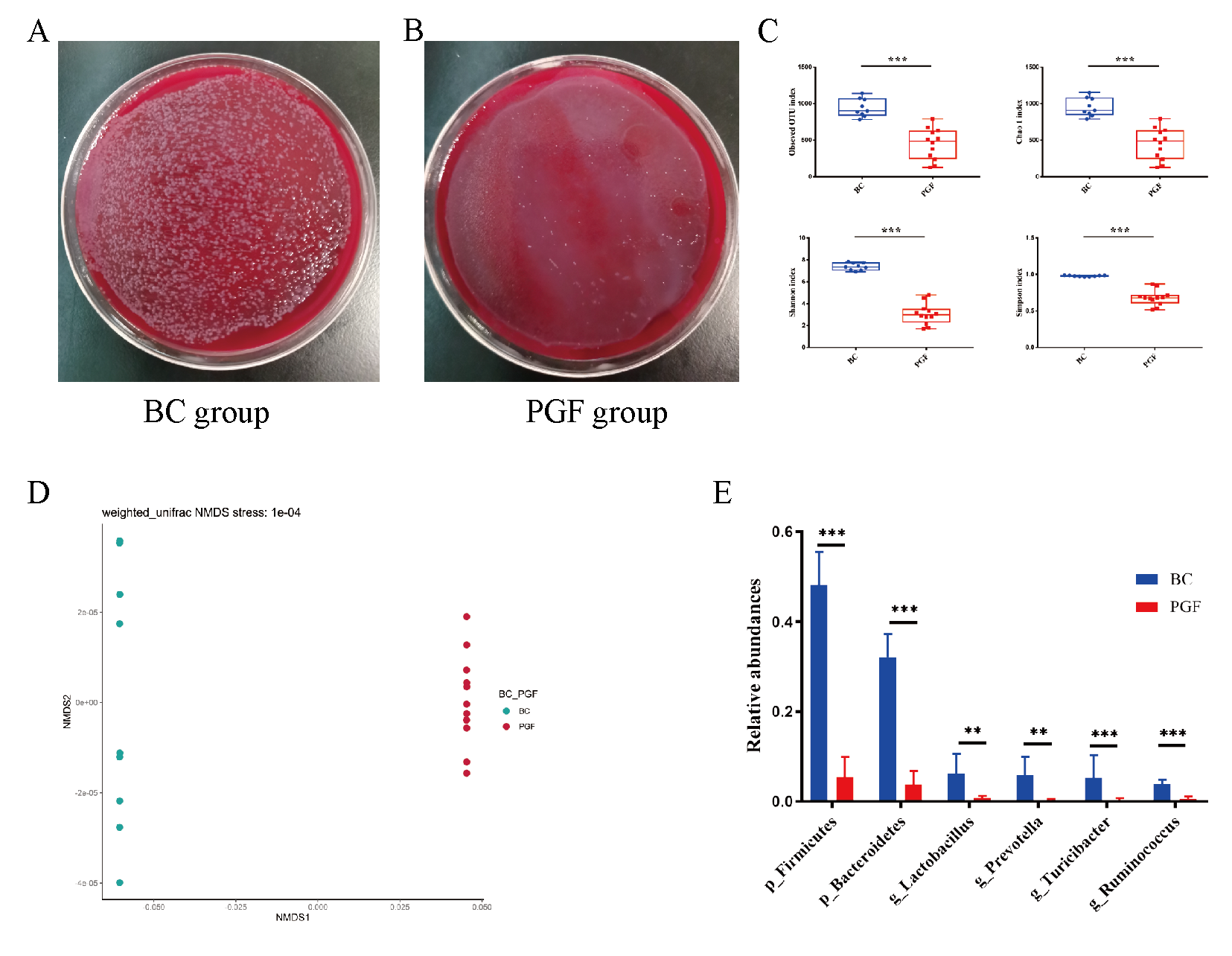

Supplement: Figure S1 — Assessment of the antibiotic cocktail-induced pseudo-germ-free rat. [file msystems.01299-23-s0001.tif]
