# Supplementary material for: The function of the gut microbiota–bile acid–TGR5 axis in diarrhea-predominant irritable bowel syndrome
Source: mSystems. 2024 Feb 8;9(3):e01299-23. doi: 10.1128/msystems.01299-23 (PMC10949424; doi:10.1128/msystems.01299-23)
Supplement: Supplemental text — Methodology of clinical section and animal experiments; legend for Fig. S1. [file msystems.01299-23-s0002.docx]

**Supplementary materials**

**1. Patients and healthy volunteers**

Diarrhea predominant-irritable bowel syndrome (IBS-D) patients were recruited through the advertisements or press released in local newspaper. The detail criteria used for patient recruitment are shown as follows:

**1.1 Inclusion criteria:** IBS-D patients were recruited on if they fulfilled the following criteria: 1) meet of Rome IV criteria, including recurrent abdominal pain on average at least 1d/week in the last 3 months; 2) appearance of stool form with at least 25% of loose or watery stools and fewer than 25% of hard stools based on Stool Bristol Score; 3) IBS Symptom Severity Scale (IBS-SSS) over than 75 points at baseline; 4) age of 18 to 70 years; 5) normal colonic evaluation with 1 years by examination of colonoscopy or barium enema; 6) Have not taken antibiotics, steroids and other hormones, Chinese herbal medicine preparations (including oral and intravenous injection) or microecological preparations in the past week; 7) Written informed consent.

**1.2 Exclusion criteria:** Patients were excluded if they have one or more of follows: 1) patients with serious heart, liver, kidney and other major organ diseases, hematopoietic system, nervous system or mental diseases; 2) combined with other organic diseases of digestive system (such as peptic ulcer), recent colonoscopy indicates organic diseases such as tumor, or systemic diseases that affect gastrointestinal motility (such as hyperthyroidism, diabetes); 3) those who are using or need to continue to use drugs that may affect gastrointestinal function (antidepressants, antidepressants, anti-anxiety drugs, intestinal flora regulators, antibiotics, etc.); 4) those who have a history of allergy to the relevant drugs under study; 5) pregnancy or breast-feeding; 6) there is currently any form of psychotherapy in progress; 7) patients who are participating in clinical trials of other drugs or within 4 weeks.

The recruiting criteria for healthy population is shown as follows:

**1.3 Inclusion criteria:** 1) age of 18–70 years (inclusive); 2) deny the existence of digestive system related symptoms such as abdominal pain, diarrhea or constipation, no other systemic organic diseases (such as cardiovascular and cerebrovascular diseases, endocrine system diseases, autoimmune diseases, etc.), no history of psychosocial diseases, and no abnormalities in the comprehensive physical examination; 3) have not taken antibiotics, steroids and other hormones, Chinese herbal medicine preparations (including oral and intravenous injection) or microecological preparations in the past week; 4) Written informed consent;

**1.4 Exclusion criteria:** 1) take antibiotics, steroids and other hormones, Chinese herbal medicine preparations (including oral and intravenous injection) or microecological preparations within one week; or use glucocorticoids, immunosuppressants, bile acid chelators, statins or other lipid-lowering drugs within three months; 2) have dysmenorrhea or other diseases that cause chronic abdominal pain; 3) pregnancy or breast-feeding; 4) history of diabetes, hyperthyroidism, chronic renal insufficiency and other diseases, combined with heart, liver, kidney, brain, hematopoietic system and other serious diseases.

**2. Establishment of** **pseudo-germ-free rats**

The clinical studies have confirmed the correlation between the gut microbiota (GM)-bile acid (BA) axis and IBS-D, but failed to reveal the mechanism of mucosal barrier damage and visceral hypersensitivity caused by abnormal GM-BA axis. To successfully carry out the subsequent fecal microbial transplantation (FMT) experiment, a pseudo-germ-free (PGF) rat model was established and evaluated. Firstly, rats were treated for four weeks with antibiotics cocktail (ABX) mixed comprising 1 g/L ampicillin (meilunbio^®^, Dalian, China), 1 g/L metronidazole (meilunbio^®^, Dalian, China), 1 g/L neomycin sulfate (MCE, New Jersey, USA) and 0.5 g/L vancomycin (meilunbio^®^, Dalian, China), and the drinking water containing antibiotics was changed twice a week[1]. Secondly, feces of rats were randomly collected for culture and 16s RNA detection.

In blank control (BC) group (prior to ABX intervention), the rat fecal diluent was inoculated on Columbia blood agar, and a large number of bacterial colonies grew after 24h anaerobic culture (Fig. S1A). In PGF group, after ABX intervention for four weeks, the rat fecal diluent was inoculated on Columbia blood agar, and there was no obvious intestinal bacterial colony growth after 24h anaerobic culture (Fig. S1B). Meanwhile, compared to baseline, 16s rRNA sequencing analysis showed a significant decrease in bacterial alpha and beta diversity in rats after ABX intervention for four weeks (Fig. S1C-D). Furthermore, at phylum level, the abundance of major bacteria such as *Firmicutes* and *Bacteroidetes* had a reduction by nearly 90%. At genus level, the abundance of major bacteria such as *Lactobacillus*, *Prevotella*, *Turicibacter*, and *Ruminococcus* had the same alternation (Fig. S1E). These results indicated PGF rat model was successfully established.

**3. Abdominal withdrawal reflex scale scores**

On the first day after inducing the models, abdominal withdrawal reflex (AWR) scale scores were used to assess colon sensitivity to colorectal distention. According to previous study[2], distention was produced by inflating a balloon inside the descending colon through the anus; the inflation balloon had four pressure grades: 20, 40, 60, and 80 mmHg. Each colorectal distention (CRD) lasted about 20 s and was repeated three times. The detailed grading rules for AWR scores were as follows: (0) no behavioral response to CRD; (1) occasional head movement at the onset of the stimulus; (2) mild abdominal muscle contraction but no lifting; (3) strong abdominal muscle contraction and the abdomen but not the pelvic structure being lifted off the platform; and (4) body arching and lifting of the pelvic structure off the platform.

**4. Detection of the moisture content of the feces**

After weighing and recording with the microbalance, they were placed in the oven (60℃) for 2 hours, and then weighed again and recorded. The ratio of the difference between the two fecal weights and the original fecal weight was recorded as the moisture content of the feces.

**5.** **Sugar water consumption test**

The rats were trained for adaptability before the test. On the first day, two bottles of the same 1% sucrose water were placed in each cage of rats for free drinking for 24 hours. On the second day, each cage was replaced with a bottle of 1% sucrose water and a bottle of purified water for free drinking for 24 hours. On the third day, the rats were deprived of food and water for 23 hours, and then were given a bottle of 1% sucrose water and a bottle of purified water of the same volume for each cage of rats to drink freely for 1 hour. Then the remaining quantities of sugar water and purified water were weighed respectively to calculate the sugar water consumption rate of rats. Sugar water consumption rate (%) = sugar water consumption / (sugar water consumption + purified water consumption) × 100%.

**6. Open field test**

At the beginning of the experiment, the rats were gently placed in a fixed position in the central area of the open field test box (100cm long, 100cm wide, 40cm high, with 16 squares of the same size on the bottom). The rats were allowed to move freely in the test box for 5 minutes and recorded the movement path in real time by the top camera. After each test, low-concentration alcohol must be used to clean the excrement of the rats at the bottom of the box. Keep the site quiet during the experiment.

**7. Electromyogram recordings of responses to colorectal distension**

After the rats were anesthetized with isoflurane, the recording electrode was placed in the external oblique muscle above the Inguinal ligament before the experimental procedure to record the Electromyography (EMG) activity. As mentioned earlier, perform CRD at the pressure of 20, 40, 60 and 80 mmHg for 20s, respectively. Rest for 2 minutes between different distension. Electromyographic (EMG) activity was recorded using Biopac Systems EMG 100c and digitized with Acknowledge. The responses were considered stable if there was less than 20% variability between 2 consecutive trials of each CRD. EMG activity was rectified, and the increase in the area under the curve (AUC) of EMG amplitude during CRD over the baseline period before CRD was recorded as the response.

**8. Small intestine propulsion rate**

Detection of small intestine propulsion rate using ink gavage method: Rats fasted for 24 hours, followed by an administration of gavage with black ink (1mL/100g). After 20 minutes, the rats were anesthetized to get the small intestine tissue. Measure the total length of the small intestine and the advance length of ink in the small intestine with a measuring stick. Small intestine propulsion rate (%) = Distance of ink propulsion in the small intestine / total length of the small intestine × 100%.

**9. Histologic, immunohistochemical, and immunofluorescence analyses**

Rat colon and spinal cord were fixed in 4% paraformaldehyde overnight at 4°C, dehydrated, soaked in xylene, embedded in paraffin in sequence, and then sliced into 4-mm sections. Paraffin sections were dewaxed with xylene and then dehydrated with different concentrations of ethanol. Sections were subjected to H&E staining and immunohistochemical as well as immunofluorescence staining. For immunohistochemistry (IHC), Primary antibody against E-cadherin (1:200, Abcam, ab181296, Cambridge, UK), CX43 (1:5000, Abcam, ab235282, Cambridge, UK), Claudin-1 (1:200, Abcam, ab15098, Cambridge, UK) and c-fos (1:100, Abcam, ab209794, Cambridge, UK) were added and incubated overnight at 4 ◦C. Following this, each slice was added secondary antibodies IgG-HRP (1:2000, Abcam, ab205718, Cambridge, UK) to combine for 30 minutes at room temperature. For immunofluorescence (IF) staining, primary antibody (1:200, Novus, NBP2-23669, Colorado, USA) was used as described for the IHC staining except for blocking endogenous peroxidase. After incubation with primary antibody, slides were incubated with secondary antibodies conjugated with a fluorochrome (Alexa Fluor® 647-conjugated goat anti-rabbit IgG (H+L), ab150079; abcam, Britain) for 1 h at room temperature in the dark.

**10. ELISA detection**

The harvested feces were used to detect the levels of Bile salt hydrolase (BSH) in rats with ELISA kits (RUXIN BIOTECH, SU-BN36884, Quanzhou, China) according to the manufacturer’s instruction. The serum of donors was used to detect the levels of FGF19 in IBS-D patients with ELISA kits (CUSABIO, CSB-EL008624HU, Wuhan, China) according to the manufacturer’s instruction.

**REFERNCE**

1. Shao S, Jia R, Zhao L, et al. Xiao-Chai-Hu-Tang ameliorates tumor growth in cancer comorbid depressive symptoms via modulating gut microbiota-mediated TLR4/MyD88/NF-kappaB signaling pathway. Phytomedicine. 2021;88:153606.

2. Wu HM, Zhan K, Rao KH, et al. Comparison of five diarrhea-predominant irritable bowel syndrome (IBS-D) rat models in the brain-gut-microbiota axis. Biomed Pharmacother. 2022;149.

**Figure legends**

Figure S1 Assessment of the antibiotic cocktail–induced (ABX-induced) pseudo-germ-free rats. (A) At baseline (prior to ABX intervention), the rat fecal diluent was inoculated on Columbia blood agar, and a large number of bacterial colonies grew after 24h anaerobic culture. (B) After ABX intervention for four weeks, the rat fecal diluent was inoculated on Columbia blood agar, and there was no obvious intestinal bacterial colony growth after 24h anaerobic culture. (C) Diversity of fecal microbial communities based on observed OTUs, Chao1, Shannon and Simpson indices. (D) Beta diversity was calculated by nonmetric multidimensional scaling based on weighted unifrac analysis. (E) Alternations of relative abundance of gut microbiota at major phyla and genus levels in rats before and after ABX-induced. Data are presented as the mean ± SD or medians with interquartile ranges. Significant differences are represented by **p* < 0.05, ***p* <0.01, ****p* <0.001, compared to the HC group. BC, Blank control; PGF, Pseudo germ-free.
