# Supplementary material for: The function of the gut microbiota–bile acid–TGR5 axis in diarrhea-predominant irritable bowel syndrome
Source: mSystems. 2024 Feb 8;9(3):e01299-23. doi: 10.1128/msystems.01299-23 (PMC10949424; doi:10.1128/msystems.01299-23)
Supplement: Table S1 — The Bristol stool form scale. [file msystems.01299-23-s0003.docx]

Table S1 The Bristol stool form scale

| Type | Characteristic |
| --- | --- |
| Type 1 | Separate hard lumps,like nuts (hard to pass) |
| Type 2 | Sausage-shaped but lumpy |
| Type 3 | Like a sausage but with cracks on its surface |
| Type 4 | Like a sausage or snake, smooth and soft |
| Type 5 | Soft blobs with clear-cut edges (passed easily) |
| Type 6 | Fluffy pieces with ragged edges, a mushy stool |
| Type 7 | Watery, no solid pieces, ENTIRELY LIQUID |
